# Multiplexed RNAs in lipid nanoparticles: potential therapeutic vaccine for chronic hepatitis B

**DOI:** 10.1038/s41392-024-02026-5

**Published:** 2024-11-07

**Authors:** Hari Krishnareddy Rachamala, Srujan Marepally

**Affiliations:** 1 Department of Biochemistry and Molecular Biology, Mayo Clinic College of Medicine and Sciences, Mayo Clinic, Jacksonville, FL 32224 USA; 2 Centre for Stem Cell Research (CSCR) (a unit of inStem, Bengaluru), CMC Campus, Vellore, 632002 TN India

**Keywords:** Molecular medicine, Nucleic-acid therapeutics

A recent study published in *Signal Transduction and Targeted Therapy* by Wenjing Z. and Min Y. et al. presents a potential game-changer in the field of chronic hepatitis B infection. The research focuses on the delivery of multiplexed RNAs using lipid nanoparticles, exploring the synergistic effects of dual small interfering RNAs (siRNAs) targeting the hepatitis B virus (HBV) alongside modulating immune responses with interleukin-2 (IL-2) mRNA.^1^

Chronic Hepatitis B (CHB) affects around 5–10% of adults and poses a higher risk in infants and children. It is indefinitely managed with nucleic acid analogs such as tenofovir and entecavir, as the functional cure is still elusive.^2^ In the recent past, the emergence of RNA interference-based approaches to silence the viral transcripts using siRNAs showed great promise in clinics in containing the virus.^3^ Multiple siRNA-based approaches, including JNJ-3989 (ARO-HBV), VIR-2218, and AB-729, are currently in clinical evaluation for the treatment of CHB. These agents directed toward silencing individual HBV transcripts achieved broad-spectrum viral suppression. Despite these promising outcomes, they could not achieve viral eradication due to their inability to eliminate covalently closed circular DNA (cccDNA), the persistent HBV reservoir. In turn, it necessitates periodic administrations, which may carry a potential risk of developing resistance over time.^2^

**Fig. 1:**
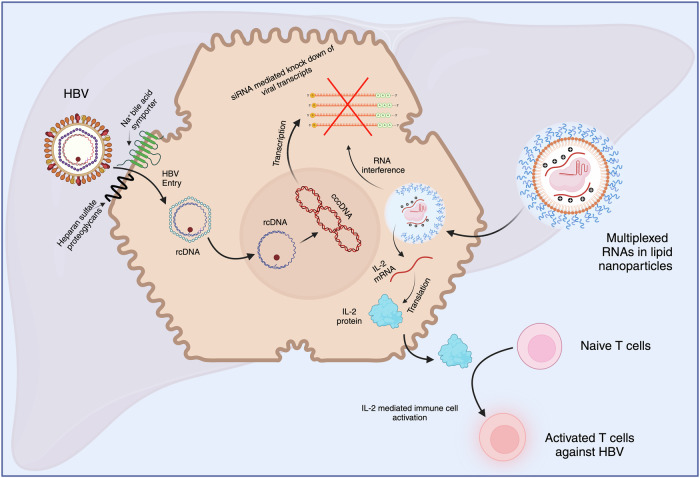
Mechanism of Hepatitis B Virus (HBV) Infection and Dual RNA-based Therapy Using siRNA and IL-2 mRNA. This figure illustrates a therapeutic strategy to target HBV by delivering multiplexed RNAs using lipid nanoparticles. The HBV enters the host cell by binding to heparan sulfate proteoglycans and is transported via the Na^+^-bile acid symporter. The viral relaxed circular DNA (crDNA) converts into covalently closed circular DNA (cccDNA) in the nucleus, acting as a viral transcription template. Small interfering RNAs (siRNAs), delivered via lipid nanoparticles, target viral transcripts for degradation through RNA interference, reducing HBV replication. Concurrently, the same nanoparticles deliver IL-2 mRNA for IL-2 protein secretion in vivo. IL-2 promotes differentiation of naive T cells into activated T cells to mount immune responses against HBV-infected cells. The figure is created with BioRender.com

To address these limitations, the study focuses on creating pan-genotypic and multifunctional siRNAs that target conserved regions of the HBV genome (Fig. 1). By targeting multiple genes or pathways, multifunctional siRNA increases the potential for effective viral silencing, even in the presence of mutations. This may effectively prevent the development of viral resistance and enhance siRNA activity, compared to targeting single genes. This is particularly important in CHB, where viral persistence and mutations are common. Targeting conserved regions across different viral strains also ensures the therapy remains effective against various HBV genotypes. Broad genotypic coverage is critical to contain multiple HBV strains spread across the globe. The multifunctional siRNAs can also produce synergistic antiviral effects, improving therapeutic outcomes while reducing the required dosages. This simplifies the treatment regimens and minimizes potential off-target effects, making the therapy safer for patients.^4^

Another aspect of the study is the choice of LNPs as the delivery vehicle for siRNA. LNPs are the preferred carriers for delivering multiple nucleic acids. LNPs can protect siRNA in systemic circulation and provide sustained release of siRNA at the target site, leading to prolonged therapeutic effects. Authors explored IL-2 mRNA to boost immune responses synergistically. IL-2 is crucial for activating and regulating the immune system. It promotes T cell proliferation and differentiation into effector cells capable of eliminating infected cells while also enhancing the cytotoxic activity of cytotoxic T lymphocytes (CTLs) and natural killer (NK) cells. Additionally, IL-2 supports B cell activation, contributing to antibody production. However, careful dosing is needed to prevent autoimmune responses. By delivering IL-2 as mRNA instead of protein, the authors leverage sustained expression and mitigate the risk of anti-IL-2 antibody generation. Additionally, mRNA delivery can be fine-tuned by adjusting stability and the dosage, allowing for more precise control over IL-2 production.^5^

In the present study, LNPs were optimized with FDA-approved ionizable lipid SM102 to increase the delivery of the siRNAs and mRNA to liver cells, thereby enhancing its therapeutic potential (Fig. 1). To assess the therapeutic potential of the siRNA candidates, 20 synthetic siRNAs were designed to target conserved motifs across the HBV genome and screened for their efficacy in RNA interference. These siRNAs were evaluated for their ability to suppress HBV replication and reduce viral antigen expression. The combination of best-performing 2 siRNAs that span 98.5% genotypic coverage was selected for further studies. These siRNAs were encapsulated in optimized LNPs with varying molar ratios of ionizable lipid, co-lipids, and HO-PEG2000-DMG lipid. The dual siRNA-loaded LNPs showed improved stability, efficacy, and safety in a preclinical setting using the rAAV-HBV1.3 mouse model. Next, these siRNAs were chemically modified with 2’-O-methyl group (2’-OMe) and 2’-Fluro (2’-F) to improve stability and efficacy. In vivo studies showed that siRNAs encapsulated LNPs efficiently reduced viral antigens and HBV DNA in single- and multiple-dose regimens. The study also revealed that combining siRNA therapy with IL-2 mRNA delivery (tLNP/siHBV-IL2) significantly reduced the viral load. This effect was achieved through two complementary mechanisms: RNAi-mediated clearance of HBsAg (Hepatitis B surface antigen) and the induction of robust HBV-specific CD4+ and CD8+ T cell responses facilitated by the expression of IL-2 protein. These findings suggest that combining siRNA and IL-2 mRNA could provide potent immune and antigenic control of HBV, paving the way for a more effective and comprehensive treatment strategy for chronic hepatitis infections.

Despite the promising findings, some areas in this research could benefit from improvement. The availability of a more appropriate animal model, which recapitulates HBV infection, is critical to assessing these therapeutics’ therapeutic efficiency with a higher precision. While the rAAV-HBV1.3 mouse model is currently available in chronic HBV infection, it has some limitations. As AAV does not integrate into the host genome, the model may not fully replicate the integrative nature of HBV in human infections. Exploring an animal model with a dual viral strategy consisting of both rAAV-HBV1.3 and a lentivirus-carrying portion of the HBV integrating genome may provide a precise representation of chronic HBV and more predictable therapeutic outcomes.

Another essential aspect to consider is the pharmacokinetic differences between siRNA and mRNA. Chemically modified siRNAs exerted their effects for about a week, while the IL-2 mRNA expression lasted 24 h. This can be overcome by tuning the structure of IL-2 mRNA to enhance stability. In a recent study, Daniel P. et al. demonstrated that fusing albumin sequences in IL2 mRNA transcript resulted in more robust cytokine expression and prolonged stability of the fusion protein for 2 days.^5^

T-cell priming was observed at relatively high mRNA dosages, which can be overcome with albumin-fused IL2 mRNA design. Reducing the dosage of mRNA while enhancing its translation efficiency could improve safety profiles.

Overall, the intriguing lipid nanoparticle-enabled approach of delivering siRNAs against 4 HBV transcripts and IL-2 mRNA synergistically inhibits the virus proliferation and efficiently primes T-cells for a superior therapeutic outcome. This therapeutic strategy not only holds promise for the treatment of chronic hepatitis B but also can be further explored for its potential to sterilizing multiple other viral infections.
